# 4418 Optimization and Validation of a Silk Scaffold-Based Neural Tissue Construct

**DOI:** 10.1017/cts.2020.85

**Published:** 2020-07-29

**Authors:** Ben Jiahe Gu, Dennis Jgamadze, Guoming (Tony) Man, Han-Chiao Isaac Chen

**Affiliations:** 1 University of Pennsylvania

## Abstract

OBJECTIVES/GOALS: Our goal is to develop a silk fibroin scaffold-based neural tissue construct and characterize it in a rat model of cortical injury. We aim to optimize the construct for transplantation, test pharmacologic interventions that may enhance its survival, and evaluate its integration with the host brain. METHODS/STUDY POPULATION: To optimize cell density and health, silk fibroin scaffolds varying in porosity and stiffness were seeded with E18 GFP+ rat cortical neurons and imaged at DIV 5. Different seeding methods and loads were similarly tested. Constructs, loaded with an inhibitor of apoptosis (ROCK inhibitor Y-27632) or necroptosis (necrostatin-1) in a fibrin hydrogel, were transplanted into aspiration lesions created in the primary motor cortex of Sprague-Dawley rats, and graft survival was compared to negative control at 2 weeks. Lastly, constructs were transplanted and evaluated via immunohistochemistry at 1, 2, and 4-month time points for survival, differentiation, inflammation, and anatomic integration. RESULTS/ANTICIPATED RESULTS: Scaffolds with smaller pore sizes retained more cells after seeding. Softer scaffolds, which enhance hemostasis at transplantation, did not compromise cell health on live/dead assay. We anticipate that seeding concentrated cell suspensions onto multiple surfaces of the construct will produce the most evenly seeded and cell-dense constructs. Based on a prior pilot study, we anticipate that necrostatin-1 will significantly improve intermediate-term construct survival. We have observed up to 15% cell survival at 1 month with retained neuronal identity and abundant axonal projections into the brain despite evidence of persistent inflammation; we anticipate similar outcomes at later time points. DISCUSSION/SIGNIFICANCE OF IMPACT: Our construct, due to its exceptional longevity in vitro, manipulability, and modularity, is an attractive platform for neural tissue engineering. In the present work, we optimize and validate this technology for transplantation with the goal of addressing the morbidity burden of cortical injury.

